# Suprachoroidal Injection of Tattoo Ink

**DOI:** 10.1177/24741264251324972

**Published:** 2025-03-14

**Authors:** Carson C. Petrash, R. Gary Lane

**Affiliations:** 1 Department of Ophthalmology, University of Texas Health at San Antonio, San Antonio, TX, USA; 2 Retina Consultants of Texas, San Antonio, TX, USA

**Keywords:** scleral tattoo, suprachoroidal injection, dye pigment

## Abstract

**Purpose:** To describe a case of injection of tattoo dye into the suprachoroidal space. **Methods:** A single case and its findings were analyzed. **Results:** A 40-year-old man with a recent history of scleral tattooing presented for evaluation of right-sided blurry vision. An examination of the right eye showed slightly decreased acuity and pupillary response in the right eye compared with the left eye as well as black ink not only in the bilateral subconjunctival spaces but also in the suprachoroidal space. Remarkably, there was no apparent inflammatory reaction. The patient was followed for 8 months after the scleral tattooing procedure and never developed inflammatory sequelae. **Conclusions:** Suprachoroidal injection of dye is a potential complication of eyeball tattooing. Infection and noninfectious inflammation are common concerns, but some patients may tolerate the dye surprisingly well.

## Introduction

Scleral tattooing is a relatively new procedure sought by clients who desire ever more extreme modifications to their appearance. Although corneal tattooing has been used legitimately to relieve photopsias associated with iris defects or to improve cosmesis of corneal scars,^1,2^ globe tattoos are strictly performed by nonophthalmic-trained individuals. Unsurprisingly, there are many reports of severe complications from scleral tattoos, whether administered in the intended subconjunctival space or erroneously injected into the periorbita or intraocular space. Other reported complications include cellulitis, scleritis, uveitis, glaucoma, cataract, retinal detachment (RD), endophthalmitis, and proliferative vitreoretinopathy.^3–6^

This case describes a unique presentation of suprachoroidal injection of tattoo ink. The findings were analyzed, including measurement of the visual acuity (VA), intraocular pressure (IOP), pupillary response, ophthalmic examination, optical coherence tomography (OCT), and fundus photography. In contrast to other reports of intraocular injection, the dye was not associated with infection or noninfectious inflammation, and the patient maintained good vision in the absence of any intervention.

## Case Report

A 40-year-old man presented with decreased vision in the right eye 11 weeks after both eyes were tattooed. He stated that during the procedure his vision went white for 5 to 7 minutes before he slowly started to see red lightning bolts. After 15 minutes, his vision had improved to approximately baseline and he subsequently elected to have the left eye tattooed as well. The procedure on the left eye was not associated with vision changes. Since then, the patient has noticed that distance vision and near vision in the right eye were blurry; however, he denied pain and sensitivity to light.

**Figure 1.**
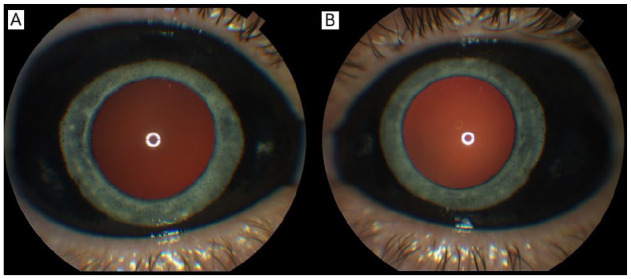
External photographs of the (A) right eye and (B) left eye show the spread of the dye throughout the bulbar conjunctiva. A darker red reflex is seen in the right eye.

The patient’s best-corrected VA was 20/30^−2^ OD and 20/20 OS. The IOP was 12 mm Hg OU. The right pupil was 3.0 mm, irregular, and sluggishly reactive to light, while the left pupil was 3.0 mm and briskly reactive. No afferent pupillary defect was observed. Notably, the Amsler grid was normal and black pigment was seen in the bulbar conjunctiva in both eyes (Figure 1).

**Figure 2.**
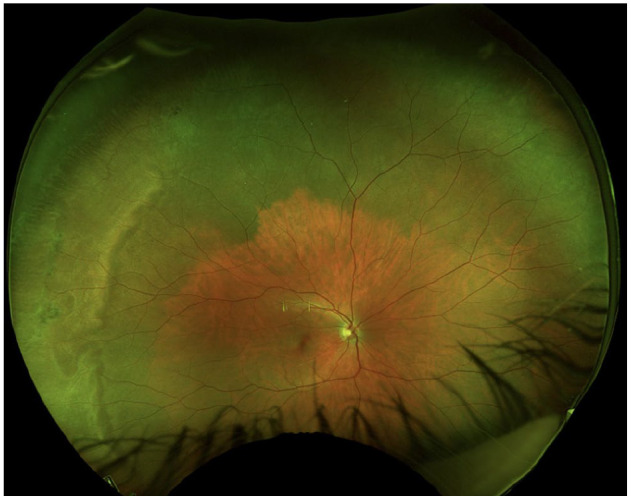
Fundus photography of the right eye shows choroidal hyperpigmentation and subretinal fibrosis. Note the remarkably clear view and the normal nerve, vessels, and macula.

**Figure 3.**
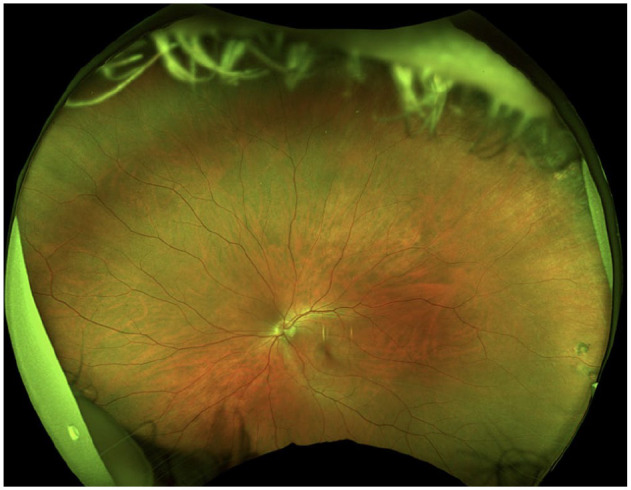
Fundus photography of the left eye shows a relatively normal fundus. There are hyperpigmented scars in the superotemporal periphery.

**Figure 4.**
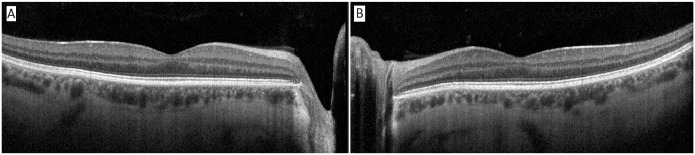
Optical coherence tomography of the (A) right macula and (B) left macula. Note the absence of macular edema or vitritis.

The anterior chamber was quiet in both eyes, and the lens and vitreous were clear. The optic nerve, macula, and retinal vasculature were normal in both eyes (Figures 2 and 3). The right eye had extensive black ink in the peripheral retina as well as subretinal fibrosis, while hyperpigmented scars were seen in the superotemporal periphery of the left eye. Although it was unclear whether the scars in the left fundus were caused by the procedure, the right eye had subretinal scars in a remarkably similar location. Furthermore, because it is possible that these scars represented trauma, the long ciliary nerve may have been injured in the right eye, contributing to the pupil’s sluggish reactivity. A normal foveal contour of the maculas was seen on OCT (Figure 4), further confirming the lack of an inflammatory reaction.

**Figure 5.**
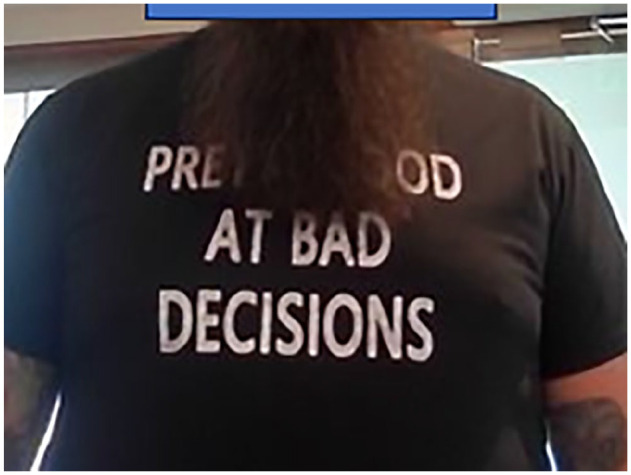
Our patient as he presented to our clinic wearing a shirt that reads, “Pretty good at bad decisions.”

The patient was aware that he was lucky to maintain vision in the right eye, as evident by his attire (Figure 5).

## Conclusions

Corneal tattooing has a long history of being used for medical and cosmetic purposes. The Roman surgeon Galen used pigments made from nuts, barks, and minerals to improve cosmesis in patients with leukocoria.^7^ In the 19th century, indications for surgery were expanded to include dysphotopsias caused by iris defects related to colobomas, traumatic iridodialysis, and albinism. Although pigments could include a range of minerals, such as copper, lead, iron, manganese, and aluminum, there was a recognition that irritating pigments should not be used and sterilization was important given that some inks could contain *Bacillus*.^7^

Today, corneal tattooing is used to improve monocular diplopia related to large iris defects as well as dysphotopsias resulting from iris transillumination defects after lateral peripheral iridotomy.^8,9^ Pigments currently in use contain platinum chloride, which is far less toxic than the minerals formerly used.^8^

Scleral tattooing to achieve dramatic appearances can be traced to a 2007 blog post in *Body Modification Ezine*, an online magazine dedicated to extreme and unusual body modifications. The original article, entitled “Three Blind Mice,” describes how the authors repeatedly struggled to inject dye into the correct tissue layer.^10^ “Probably about forty strikes in all were done,” with one “potentially entering the sclera itself.” Photographs featured in the article indicate that the sclera was penetrated. The author finishes with, “Please wait for us to either heal or go blind before trying it.” Although this recklessness is unfathomable to most ophthalmologists, this quote shows the state of mind of patients who volunteer for these types of “experiments.”

Fortunately, fewer than 20 cases of scleral tattooing have been described in the literature. Without penetration of the globe, dye toxicity is limited to conjunctivitis or scleritis and may even dissipate over time.^11^ Most published cases, however, are associated with penetration of the globe, which leads to complications including endophthalmitis, uveitis, RD, glaucoma, cataract, and corneal failure.^11^

To our knowledge, no previous reports have described intraocular injection of tattoo ink without severe inflammatory reaction, either infectious or toxicity from mineral components. Our patient’s small decrease in VA, whether it is attributable to the dye injection, is a remarkably limited response to this foreign body. Black tattoo dyes are typically carbon based and do not contain heavy metals. Although the content of the dye was not able to be ascertained in this case, and given the lack of an inflammatory reaction, it is likely that it was carbon (not mineral) based. Furthermore, the subretinal space and suprachoroidal space have some degree of immune privilege, lacking native immune cells and lymphatics and subsequently limiting the inflammatory response to dye. Whether this played a role in this case is not known but is plausible. Our patient was extremely fortunate or, in his words, “Pretty good at bad decisions” (Figure 5). We hope this trend is recognized as dangerous and diminishes in popularity.
